# 5,5′-(Butane-1,4-di­yl)bis­(1*H*-tetra­zole) dihydrate

**DOI:** 10.1107/S1600536810048993

**Published:** 2010-11-27

**Authors:** Jian-Hua Xin, Xiao-Lan Tong

**Affiliations:** a College of Biology, Chemistry and Material Science, East China of Technology, 344000 Fuzhou, Jiangxi, People’s Republic of China

## Abstract

The title compound, C_6_H_10_N_8_·2H_2_O, was prepared by the reaction of hexanedinitrile and sodium azide. The di-1*H*-tetra­zole mol­ecule lies on a crystallographic centre of inversion and is linked to the water mol­ecules by N—H⋯O and O—H⋯N hydrogen bonds, forming a two-dimensional supra­molecular structure in the crystal.

## Related literature

For tetra­zole derivatives, see: Demko & Sharpless (2001 ▶); Diop *et al.* (2002 ▶); Kitagawa *et al.* (2004 ▶); Li *et al.* (2007 ▶); Tamura *et al.* (1998 ▶); Tong *et al.* (2009 ▶); Zhao *et al.* (2008 ▶).
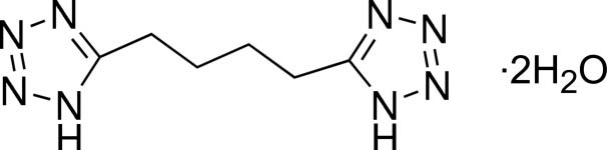


## Experimental

### 

#### Crystal data

C_6_H_10_N_8_·2H_2_O
*M* *_r_* = 230.25
Monoclinic, 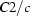

*a* = 6.994 (3) Å
*b* = 11.590 (5) Å
*c* = 14.097 (6) Å
β = 100.716 (7)°
*V* = 1122.8 (8) Å^3^
*Z* = 4
Mo *K*α radiation
μ = 0.11 mm^−1^
*T* = 294 K
0.20 × 0.18 × 0.16 mm

#### Data collection

Bruker SMART CCD area-detector diffractometer
Absorption correction: multi-scan (*SADABS*; Sheldrick, 1996 ▶) *T*_min_ = 0.979, *T*_max_ = 0.983
2756 measured reflections
992 independent reflections
722 reflections with *I* > 2σ(*I*)
*R*_int_ = 0.025

#### Refinement

*R*[*F* ^2^ > 2σ(*F* ^2^)] = 0.052
*wR*(*F* ^2^) = 0.147
*S* = 1.04
992 reflections
73 parameters
H-atom parameters constrained
Δρ_max_ = 0.16 e Å^−3^
Δρ_min_ = −0.21 e Å^−3^

### 

Data collection: *SMART* (Siemens, 1996 ▶); cell refinement: *SAINT* (Siemens, 1996 ▶); data reduction: *SAINT*; program(s) used to solve structure: *SHELXS97* (Sheldrick, 2008 ▶); program(s) used to refine structure: *SHELXL97* (Sheldrick, 2008 ▶); molecular graphics: *DIAMOND* (Brandenburg, 1999 ▶); software used to prepare material for publication: *SHELXL97*.

## Supplementary Material

Crystal structure: contains datablocks global, I. DOI: 10.1107/S1600536810048993/bt5418sup1.cif

Structure factors: contains datablocks I. DOI: 10.1107/S1600536810048993/bt5418Isup2.hkl

Additional supplementary materials:  crystallographic information; 3D view; checkCIF report

## Figures and Tables

**Table 1:** Hydrogen-bond geometry (Å, °)

*D*—H⋯*A*	*D*—H	H⋯*A*	*D*⋯*A*	*D*—H⋯*A*
O1*W*—H1*WB*⋯N3^i^	0.85	2.02	2.851 (3)	165
O1*W*—H1*WA*⋯N4^ii^	0.85	1.99	2.822 (3)	167
N1—H1⋯O1*W*	0.86	1.80	2.662 (3)	175

Symmetry codes: (i) 
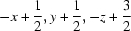
; (ii) 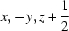.

**Fig. 1.**
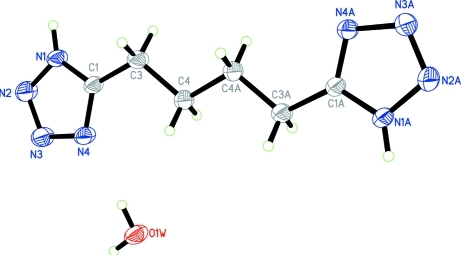
The asymmetric unit of the title compound, (I), with displacement ellipsoids drawn at the 30% probability level.

**Fig. 2.**
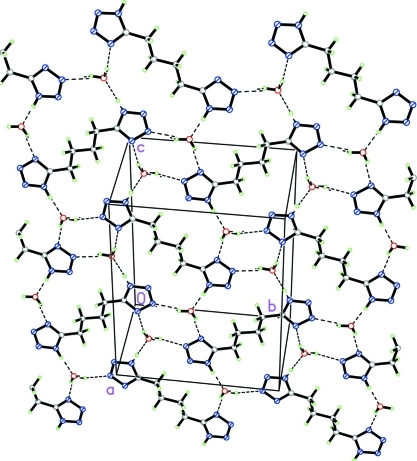
The packing diagram of the title compound. Hydrogen bonds are shown as dashed line.

## supplementary crystallographic information

### Comment

The tetrazole derivatives are very important molecules in pharmacological and biochemical properties (Tamura *et al.*, 1998). Since Sharpless *et al.* have introduced a simple and effective method to synthesize the tetrazole derivatives (Demko *et al.*, 2001), they have been used extensively in areas as diverse as medicinal chemistry, coordination chemistry and material chemistry (Zhao *et al.*, 2008; Kitagawa *et al.*, 2004; Li *et al.*, 2007). Among these, The flexible 5-substituted tetrazolate ligands have been less investigated (Diop *et al.*, 2002), although we have studied the coordination of the bis(tetrazole) ligands separated by alkyl (CH_2_)_n_ spacers (Tong *et al.*, 2009). Here, as the additional of our work, we report the crystal structure of the title compound (Fig. 1).

1,2-Bis(tetrazol-5-yl)butpane lies on a crystallographic centre of inversion and is linked to the water molecules by N—H···O and O—H···N hydrogen bonds into a 2-D supramolecular structure (Fig. 2).

### Experimental

1,2-Bis(tetrazol-5-yl)butane was prepared using a reported procedure (Tong *et al.*, 2009) (Scheme I). 1,2-Bis(tetrazol-5-yl)butane and water (12 ml) was sealed in a 25 ml Teflon-lined stainless steel vessel and heated at 393 k for 72 hr., then cooled to room temperature. Colorless prism-shaped crystals of the title compound were isolated and washed with water and ethanol and dried in air.

### Refinement

All H atoms were placed in idealized positions (O—H = 0.85 Å, N—H = 0.86 Å and C—H = 0.95 Å) and refined as riding atoms with *U*_iso_(H) = 1.2*U*_eq_(C, N) and *U*_iso_(H) = 1.5*U*_eq_(O).

### Crystal data

**Table:**

C_6_H_10_N_8_·2H_2_O	*F*(000) = 488
*M**_r_* = 230.25	*D*_x_ = 1.362 Mg m^−^^3^
Monoclinic, *C*2/*c*	Mo *K*α radiation, λ = 0.71073 Å
Hall symbol: -C 2yc	Cell parameters from 1178 reflections
*a* = 6.994 (3) Å	θ = 2.9–25.0°
*b* = 11.590 (5) Å	µ = 0.11 mm^−^^1^
*c* = 14.097 (6) Å	*T* = 294 K
β = 100.716 (7)°	Block, colorless
*V* = 1122.8 (8) Å^3^	0.20 × 0.18 × 0.16 mm
*Z* = 4	

### Data collection

**Table:**

Bruker SMART CCD area-detector diffractometer	992 independent reflections
Radiation source: fine-focus sealed tube	722 reflections with *I* > 2σ(*I*)
graphite	*R*_int_ = 0.025
φ and ω scans	θ_max_ = 25.0°, θ_min_ = 2.9°
Absorption correction: multi-scan (*SADABS*; Sheldrick, 1996)	*h* = −7→8
*T*_min_ = 0.979, *T*_max_ = 0.983	*k* = −13→10
2756 measured reflections	*l* = −16→15

### Refinement

**Table:**

Refinement on *F*^2^	Primary atom site location: structure-invariant direct methods
Least-squares matrix: full	Secondary atom site location: difference Fourier map
*R*[*F*^2^ > 2σ(*F*^2^)] = 0.052	Hydrogen site location: inferred from neighbouring sites
*wR*(*F*^2^) = 0.147	H-atom parameters constrained
*S* = 1.03	*w* = 1/[σ^2^(*F*_o_^2^) + (0.0759*P*)^2^ + 0.9248*P*] where *P* = (*F*_o_^2^ + 2*F*_c_^2^)/3
992 reflections	(Δ/σ)_max_ < 0.001
73 parameters	Δρ_max_ = 0.16 e Å^−^^3^
0 restraints	Δρ_min_ = −0.21 e Å^−^^3^

### Special details

**Table:**

Geometry. All e.s.d.'s (except the e.s.d. in the dihedral angle between two l.s. planes) are estimated using the full covariance matrix. The cell e.s.d.'s are taken into account individually in the estimation of e.s.d.'s in distances, angles and torsion angles; correlations between e.s.d.'s in cell parameters are only used when they are defined by crystal symmetry. An approximate (isotropic) treatment of cell e.s.d.'s is used for estimating e.s.d.'s involving l.s. planes.
Refinement. Refinement of *F*^2^ against ALL reflections. The weighted *R*-factor *wR* and goodness of fit *S* are based on *F*^2^, conventional *R*-factors *R* are based on *F*, with *F* set to zero for negative *F*^2^. The threshold expression of *F*^2^ > σ(*F*^2^) is used only for calculating *R*-factors(gt) *etc*. and is not relevant to the choice of reflections for refinement. *R*-factors based on *F*^2^ are statistically about twice as large as those based on *F*, and *R*- factors based on ALL data will be even larger.

### Fractional atomic coordinates and isotropic or equivalent isotropic displacement parameters (Å^2^)

**Table:**

	*x*	*y*	*z*	*U*_iso_*/*U*_eq_	
O1W	0.2175 (3)	0.10913 (15)	0.88483 (13)	0.0697 (7)	
H1WA	0.2503	0.0847	0.9424	0.105*	
H1WB	0.2153	0.1823	0.8886	0.105*	
N1	0.2521 (3)	−0.00531 (16)	0.72515 (14)	0.0487 (6)	
H1	0.2430	0.0355	0.7751	0.058*	
N2	0.2517 (4)	−0.11987 (17)	0.72219 (16)	0.0607 (7)	
N3	0.2679 (4)	−0.14652 (18)	0.63566 (16)	0.0624 (7)	
N4	0.2787 (4)	−0.05060 (17)	0.58246 (14)	0.0536 (7)	
C1	0.2683 (4)	0.0365 (2)	0.63995 (16)	0.0422 (6)	
C3	0.2750 (4)	0.1611 (2)	0.61690 (17)	0.0505 (7)	
H3A	0.3991	0.1922	0.6488	0.061*	
H3B	0.1737	0.2006	0.6428	0.061*	
C4	0.2488 (4)	0.1863 (2)	0.50977 (17)	0.0463 (7)	
H4A	0.3525	0.1493	0.4838	0.056*	
H4B	0.1261	0.1541	0.4771	0.056*	

### Atomic displacement parameters (Å^2^)

**Table:**

	*U*^11^	*U*^22^	*U*^33^	*U*^12^	*U*^13^	*U*^23^
O1W	0.131 (2)	0.0449 (11)	0.0368 (11)	0.0010 (11)	0.0256 (11)	−0.0017 (8)
N1	0.0817 (16)	0.0361 (12)	0.0306 (11)	−0.0005 (10)	0.0162 (10)	0.0006 (9)
N2	0.101 (2)	0.0406 (13)	0.0419 (13)	−0.0030 (12)	0.0169 (12)	0.0072 (10)
N3	0.108 (2)	0.0365 (12)	0.0451 (14)	−0.0027 (12)	0.0196 (13)	0.0011 (10)
N4	0.0960 (18)	0.0327 (11)	0.0344 (11)	−0.0022 (11)	0.0181 (11)	0.0010 (9)
C1	0.0603 (16)	0.0364 (12)	0.0306 (12)	−0.0024 (11)	0.0103 (10)	0.0003 (10)
C3	0.082 (2)	0.0344 (13)	0.0373 (14)	−0.0022 (12)	0.0157 (12)	−0.0011 (11)
C4	0.0661 (16)	0.0366 (13)	0.0373 (13)	−0.0007 (12)	0.0120 (11)	0.0020 (10)

### Geometric parameters (Å, °)

**Table:**

O1W—H1WA	0.8500	C1—C3	1.482 (3)
O1W—H1WB	0.8500	C3—C4	1.516 (3)
N1—C1	1.320 (3)	C3—H3A	0.9700
N1—N2	1.328 (3)	C3—H3B	0.9700
N1—H1	0.8600	C4—C4^i^	1.503 (5)
N2—N3	1.284 (3)	C4—H4A	0.9700
N3—N4	1.351 (3)	C4—H4B	0.9700
N4—C1	1.305 (3)		
			
H1WA—O1W—H1WB	106.1	C1—C3—H3A	108.8
C1—N1—N2	109.8 (2)	C4—C3—H3A	108.8
C1—N1—H1	125.1	C1—C3—H3B	108.8
N2—N1—H1	125.1	C4—C3—H3B	108.8
N3—N2—N1	105.69 (19)	H3A—C3—H3B	107.7
N2—N3—N4	110.7 (2)	C4^i^—C4—C3	111.7 (3)
C1—N4—N3	106.1 (2)	C4^i^—C4—H4A	109.3
N4—C1—N1	107.7 (2)	C3—C4—H4A	109.3
N4—C1—C3	127.6 (2)	C4^i^—C4—H4B	109.3
N1—C1—C3	124.7 (2)	C3—C4—H4B	109.3
C1—C3—C4	113.8 (2)	H4A—C4—H4B	108.0
			
C1—N1—N2—N3	0.1 (3)	N2—N1—C1—N4	−0.1 (3)
N1—N2—N3—N4	0.0 (3)	N2—N1—C1—C3	−179.6 (2)
N2—N3—N4—C1	0.0 (3)	N4—C1—C3—C4	13.6 (4)
N3—N4—C1—N1	0.1 (3)	N1—C1—C3—C4	−167.1 (3)
N3—N4—C1—C3	179.5 (2)	C1—C3—C4—C4^i^	178.4 (3)

Symmetry codes: (i) −*x*+1/2, −*y*+1/2, −*z*+1.

### Hydrogen-bond geometry (Å, °)

**Table:**

*D*—H···*A*	*D*—H	H···*A*	*D*···*A*	*D*—H···*A*
O1W—H1WB···N3^ii^	0.85	2.02	2.851 (3)	165
O1W—H1WA···N4^iii^	0.85	1.99	2.822 (3)	167
N1—H1···O1W	0.86	1.80	2.662 (3)	175

Symmetry codes: (ii) −*x*+1/2, *y*+1/2, −*z*+3/2; (iii) *x*, −*y*, *z*+1/2.
